# A robust nonlinear low-dimensional manifold for single cell RNA-seq data

**DOI:** 10.1186/s12859-020-03625-z

**Published:** 2020-07-21

**Authors:** Archit Verma, Barbara E. Engelhardt

**Affiliations:** 1 Chemical and Biological Engineering, Princeton University, 50-70 Olden Street, Princeton, 08540 NJ USA; 2 Computer Science, Center for Statistics and Machine Learning, 35 Olden Street, Princeton, 08540 NJ USA

**Keywords:** Manifold learning, Single cell RNA sequencing, Gaussian process latent variable model, Dimension reduction, Robust model, Nonlinear maps

## Abstract

**Background:**

Modern developments in single-cell sequencing technologies enable broad insights into cellular state. Single-cell RNA sequencing (scRNA-seq) can be used to explore cell types, states, and developmental trajectories to broaden our understanding of cellular heterogeneity in tissues and organs. Analysis of these sparse, high-dimensional experimental results requires dimension reduction. Several methods have been developed to estimate low-dimensional embeddings for filtered and normalized single-cell data. However, methods have yet to be developed for unfiltered and unnormalized count data that estimate uncertainty in the low-dimensional space. We present a nonlinear latent variable model with robust, heavy-tailed error and adaptive kernel learning to estimate low-dimensional nonlinear structure in scRNA-seq data.

**Results:**

Gene expression in a single cell is modeled as a noisy draw from a Gaussian process in high dimensions from low-dimensional latent positions. This model is called the Gaussian process latent variable model (GPLVM). We model residual errors with a heavy-tailed Student’s t-distribution to estimate a manifold that is robust to technical and biological noise found in normalized scRNA-seq data. We compare our approach to common dimension reduction tools across a diverse set of scRNA-seq data sets to highlight our model’s ability to enable important downstream tasks such as clustering, inferring cell developmental trajectories, and visualizing high throughput experiments on available experimental data.

**Conclusion:**

We show that our adaptive robust statistical approach to estimate a nonlinear manifold is well suited for raw, unfiltered gene counts from high-throughput sequencing technologies for visualization, exploration, and uncertainty estimation of cell states.

## Background

High-throughput single-cell RNA sequencing (scRNA-seq) is a powerful tool for cataloguing cell types and cell states, and for investigating changes in expression over cell developmental trajectories. Droplet-based methods encapsulate individual cells with unique barcode tags that are ligated to cellular RNA fragments [1]. Sequenced reads are mapped to both a gene and a cell, creating a high-dimensional cell-by-gene count matrix with hundreds to millions of cells and twenty thousand genes per human cell. These cell-by-gene count matrices contain a substantial proportion of zeros because of low-coverage sequencing per cell (i.e., *dropout*). The count matrices also contain substantial variance and observation outliers due to both technical and biological sources of noise [2]. Furthermore, the low-dimensional space representing the transcriptional relationships among cells is not a simple, smooth space, but a complex, nonlinear, and sometimes non-smooth space due to cellular differentiation and cell cycle processes.

Computational tools for analyzing scRNA-seq results generally require initial dimension reduction to a lower-dimensional manifold capturing gene expression patterns for regularization and computational efficiency. Dimension reduction techniques are an essential precursor to noise reduction [2, 3], sub-population identification [4, 5], visualization [6, 7], pseudotemporal ordering of development stages [8–10], and imputation [11]. Lower-dimensional mappings also provide convenient visualizations that lead to hypothesis generation, and inform analytic methods and future experiments [12, 13].

Linear dimension reduction techniques are commonly used as a first step to downstream analyses. Principal component analysis [14] (PCA) – the projection of a high-dimensional space onto orthogonal bases that capture the directions of greatest variance – is the first step of several scRNA-seq analysis packages such as PAGODA [15] and Waterfall [16]. Zero-Inflated Factor Analysis [4] (ZIFA) extends the factor analysis [17] paradigm of a linear map onto low-dimensional latent dimensions to allow dropouts modeled by Bernoulli random variables to account for the excess of zero counts in scRNA-seq data. Independent component analysis [18] (ICA), which assumes non-Gaussian observations, and canonical correlation analysis [19] (CCA), which allows for multiple observation types, have also been used as dimension reduction techniques for studying cell developmental trajectories [9] and for experimental batch correction [20].

More sophisticated models eschew the linearity assumption to capture the rich nonlinear structure in the data. The t-distributed Stochastic Neighbors Embedding (t-SNE) [7] is a popular visualization tool. T-SNE computes the similarity between two points in high-dimensional space with respect to a Gaussian kernel distance metric, and estimates a lower-dimensional mapping with similarity with respect to a Student’s t-distribution metric that minimizes the Kullback-Leibler divergence between the similarity distributions in high and low dimensions. The Gaussian kernel in t-SNE includes a perplexity parameter that controls the decay rate of similarity across the distance between cells. The uniform manifold approximation and projection for dimension reduction [21] (UMAP), which is similar to t-SNE but better preserves high-dimensional distances in the loudimentioned space, has also become popular for visualization. Diffusion maps, used in packages such as Destiny [22], are another tool for nonlinear low-dimensional mapping that perform linear decomposition on a kernel similarity matrix of high-dimensional observations. SAUCIE [6] implements an autoencoder, or a deep neural network, that compresses data with the goal of creating an optimal reconstruction from the compressed representation in order to execute several single-cell tasks. Similarly, scVI [23] uses deep neural networks to create a probabilistic representation in latent space for batch correction, visualization, clustering, and differential expression. One Bayesian probabilistic technique is the Gaussian process latent variable model [24, 25] (GPLVM), used by scLVM [2] for noise reduction, and GPfates [10] and GrandPrix [8] for pseudotemporal ordering. The GPLVM models observations (i.e., cells) as draws from a Gaussian process map of lower-dimensional latent variables.

While current methods for dimension reduction have been successful with early sequencing experiments and filtered expression data, they are limited in their capacity to accurately represent and inform analyses of raw, high-throughput sequencing experiments. Linear methods such as PCA and ZIFA are ill-suited for capturing highly nonlinear biological processes across developmental phase, and many implementations scale poorly with increased sample size. Current nonlinear methods are highly sensitive to parameter choices, including perplexity for t-SNE, kernel variables for diffusion maps, and network architecture for VAEs. Latent dimensions of t-SNE have no global structure, making embedded positions difficult to interpret and leading to uninformative mappings beyond two dimensions. Downstream analyses of t-SNE results are hindered by an inability to map the low-dimensional projections back to observation space [26]. VAEs, like most neural networks, require tens to hundreds of thousands of cells for accurate estimation, which may not be available in smaller experiments. Furthermore, VAEs are often challenging to fit in practice, with *posterior collapse* being a common failure mode in statistical inference [27]. These current methods, particularly those using the GPLVM, are sensitive to outliers. In particular, they often work only with filtered, normalized data, and they incorporate prior information to facilitate the latent mapping.

Robust statistical models are a natural solution to capturing heavy-tailed, sparse log count data. We introduce the t-distributed Gaussian process latent variable model (tGPLVM) for learning a low-dimensional, nonlinear embedding of unfiltered count data. To address each of the challenges in scRNA-seq analyses, we introduce three features to the basic GPLVM: 1) a robust Student’s t-distribution noise model; 2) a weighted sum of non-smooth covariance kernel functions with parameters estimated from the data; 3) sparse kernel structure. The heavy tailed Student’s t-distribution improves robustness to outliers, previously demonstrated in Gaussian process regression [28, 29]. Matérn kernels have been successfully used in single-cell time series models to capture non-smooth trajectories [8, 30, 31]. We use a weighted sum of three Matérn kernels (1/2, 3/2, and 5/2) and a Gaussian kernel. We estimate the kernel parameters during inference to allow for a broad range of high-dimensional geometries. The sparse kernel structure allows us to effectively reduce the number latent dimensions based on the estimated complexity of the data. Furthermore, our implementation of tGPLVM accepts sparse inputs produced from high-throughput experimental cell-by-gene count matrices, is among the best methods in terms of computational speed, and scales to over a million cells.

We demonstrate tGPLVM’s ability to estimate informative manifolds from noisy, raw single-cell unique molecular identifier (UMI) count matrices and highlight its applicability to multiple downstream tasks. While still sparse, UMIs do not show zero-inflation and require less processing or normalization than traditional count data [32]. We show improved cell-type identification via clustering on the estimated latent space using a data set of cerebral cortex cells labeled with estimated cell type [33]. We find that the tGPLVM manifold can learn pseudotemporal ordering from a batch of *Plasmodium*-infected mouse cells sequenced across time post exposure [10]. Finally, we demonstrate that tGPLVM can be used on unprocessed, unnormalized count data from recent high-throughput sequencing methods [1] and can be used to explore gene expression across cell states.

## Results

The t-distributed Gaussian process latent variable model (tGPLVM) is a nonlinear latent variable model that captures high-dimensional observations in a low-dimensional nonlinear latent space. Expression of each of *P* genes across *N* cells is modeled as a draw from a Gaussian process distribution with the covariance matrix a function of the low-dimensional, latent positions. The observation error is modeled with a heavy-tailed Student’s t-distribution with four degrees of freedom to robustly account for variance in the count data due to technical and biological noise relative to a normally distributed error model [34, 35]. Here, we used a weighted sum of Matérn 1/2, Matérn 3/2, Matérn 5/2, and squared exponential (SE) kernel functions to model possibly non-smooth manifolds.

### Student’s t-distributed error improves manifold learning

**Fig. 1:**
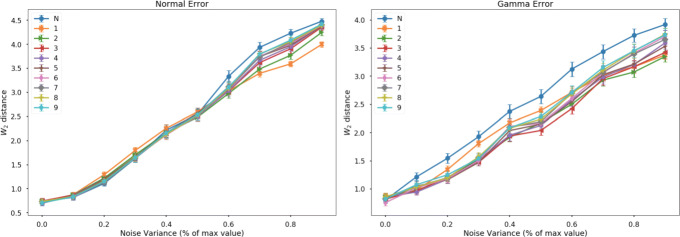
Average Wasserstein-2 distance across samples of the normalized pairwise distance matrix between learned manifold and true manifolds as a function of noise variance for (left) Gaussian error model and (right) zero-centered gamma error model. Numbers indicate the Student’s t-distribution degrees of freedom parameters, N represent normal error. Error bars represent the standard error of the mean

To evaluate the impact of using a Student’s t-distributed error model instead of a Gaussian, we compared the accuracy of estimated latent representations of simulated data. Data from a branching trajectory in two dimensions are projected into ten-dimensional space by a SE kernel Gaussian process. We added noise to the simulation with either Gaussian or gamma distributions, then fit using either t-distributed or Gaussian error GPLVMs. We evaluated the goodness-of-fit by comparing the 2-Wasserstein distance between normalized pairwise distance matrices of the simulated and estimated latent positions. For simulations with normally distributed error, t-distributed and normal error models are about equal in their ability for reconstruct the latent space (Fig. 1). As the variance of the Gaussian error increases past 50% of the maximum noiseless value, however, the t-distributed model outperforms a normally distributed error model. When the noise is a skewed gamma distribution, using the t-distributed error is uniformly better than the normal distribution at estimating the latent space. When the data-generating covariance is different from the model covariance, heavy-tailed noise models deal well with model mismatch. These results demonstrate that a robust Student’s t-distributed error model improves estimation under different possible noise conditions.

### Nonparametric manifold learning improves cell-type identification.

**Fig. 2:**
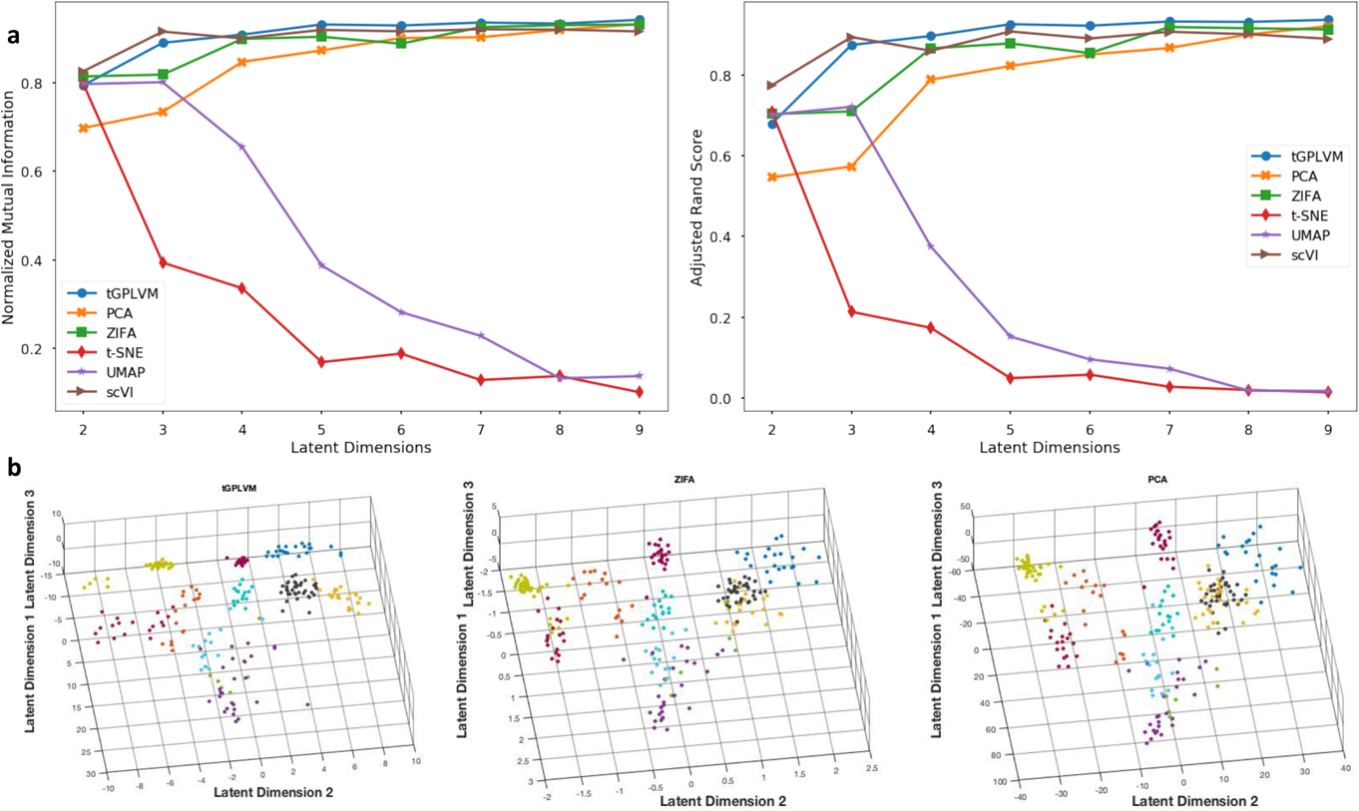
Comparison of manifold learning methods on 11 neural and blood cell populations [33]. **a** Average adjusted rand score (ARS; left) and normalized mutual information (NMI; right) of ten k-means cluster labels versus available cell type labels with respect to the number of latent dimensions. **b** Three dimensional latent mappings from tGPLVM, PCA, and ZIFA colored by inferred cell type label. t-SNE (not pictured) collapses in three dimensions

We evaluated the ability of tGPLVM and commonly-used single-cell dimension reduction methods to distinguish distinct cell types. tGPLVM, PCA, ZIFA, t-SNE, UMAP, and scVI were used to map cells labeled with their inferred cell type from the Pollen data [33] to latent spaces varying from two to nine dimensions. The data consist of log _10_(1+*x*) normalized counts of 249 cells from 11 distinct populations from a Fluidigm C1 system. With more than three latent dimensions, tGPLVM produced clusters that best corresponded to the actual cell-type labels of the six methods (Fig. 2). With three dimensions, tGPVLM is second only to scVI in performance. At nine latent dimensions, the models perform about equally, indicating that most of the information in the data has been captured.

**Fig. 3:**
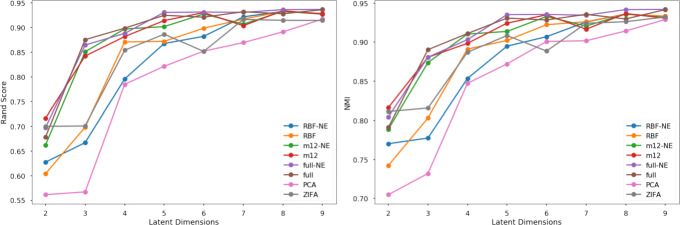
Comparison of kernel and error models on 11 neural and blood cell populations [33]: Average adjusted rand score (left) and normalized mutual information (right) of ten k-means cluster labels against true cell type labels versus number of latent dimensions learned (legend) NE - normal error model, RBF - radial basis function kernel only, m12 - sum of RBF and Matérn 1/2 kernels, full - sum of RBF, Matérn 1/2, Matérn 3/2, and Matérn 5/2 kernels

**Fig. 4:**
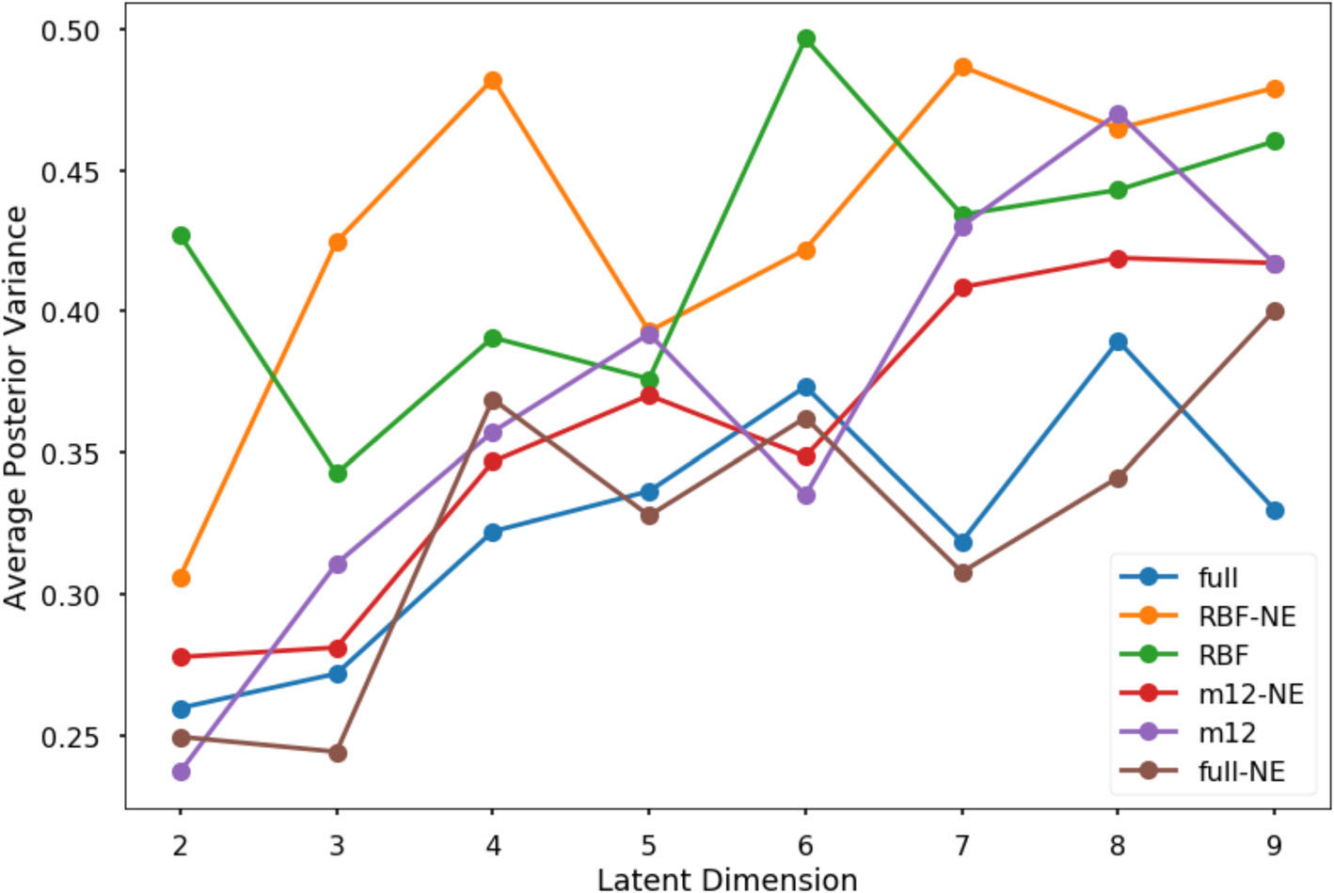
Average posterior variance of kernel and error models on 11 neural and blood cell populations [33]: Average posterior variance of the distribution of latent positions (legend) NE - normal error model, RBF - radial basis function kernel only, m12 - sum of RBF and Matérn 1/2 kernels, full - sum of RBF, Matérn 1/2, Matérn 3/2, and Matérn 5/2 kernels

Including Matérn kernels in tGPLVM improves cell-type separation in the latent space as measured by normalized mutual information (NMI) and adjusted rand score (ARS) (Fig. 3). Inclusion of Matérn kernels also reduces the uncertainty of posterior estimates of the latent embedding as measured by the average scale parameter of the latent position (Fig. 4). We find that the clustering efficacy remains when initialization is changed from a PCA approximation to random initial embeddings (Supplemental Figure 1). These results suggest that a robust Bayesian nonparametric manifold is superior to current linear dimension reduction algorithms and equal to scVI for identifying and visualizing distinct cell types captured by scRNA-seq experiments.

### Nonparametric manifolds to reconstruct development time scales without prior information

**Fig. 5:**
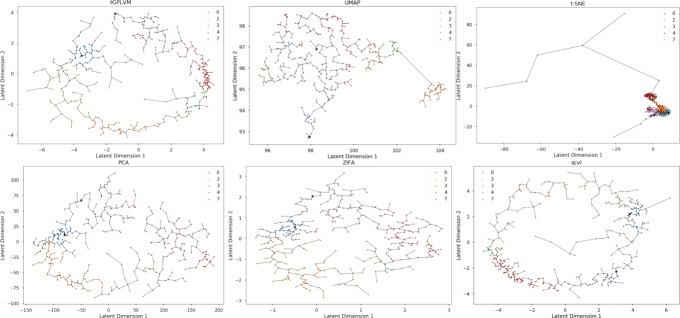
Comparison of manifold learning Methods on *Plasmodium*-infected Th1 and Tfh cells [10]: Plot of two dimensional latent mapping from tGPLVM, PCA, ZIFA, and t-SNE. Labels indicate days after infection prior to sequencing. Dotted lines represent connections along the minimum spanning tree

Next, we test the flexibility of tGPLVM applied to continuous cellular developmental trajectories. We fit latent mappings for log count data of 408 mouse Th1 and Tfh cells sequenced over seven days on a Fluidigm C1 after infection with *Plasmodium* [10]. Visually, we find that the latent mapping from tGPLVM represents the temporal relationships accurately, with most cells positioned among cells from the same or adjacent time points. We build a minimum spanning tree on the latent mappings to infer developmental trajectories. For a two-dimensional mapping, only tGPLVM accurately spans the first time point (day 0) to the final time point (day 7). PCA, ZIFA, and scVI find endpoints of the tree in days 2 or 4. t-SNE is able to separate cells based on time but does not accurately reconstruct the ordering and is sensitive to outliers (Fig. 5). UMAP’s representation does not represent the time progression as well as other methods. We recognize that these trees are not a complete method for determining developmental trajectories, and that PCA, ZIFA, scVI, and tGPLVM are all capable of finding reasonable embeddings. The results suggest that tGPLVM can capture developmental trajectories and distances in unlabeled settings better than other visualization tools and as well as any other single-cell dimension reduction technique.

### Nonparametric manifold learning improves visualization of raw count data and captures cell state

**Fig. 6:**
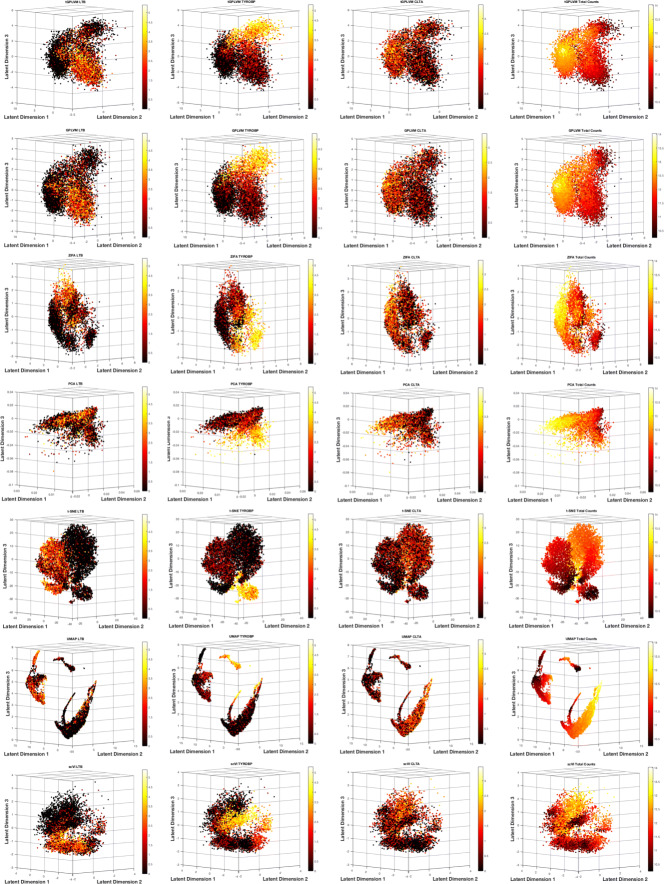
Manifold learning methods on unprocessed CD34+ PBMCs [1] counts. tGPLVM shows the best separation of expression patterns based on cell state marker genes. Color bars indicate log2(1+*Y*), where *Y* represents total counts across genes and cells

Next, we tested tGPLVM’s performance on unfiltered count data. tGPLVM and comparison models were fit on ∼10,000 CD34+ peripheral blood mononuclear cells (PBMCs) sequenced on a high-throughput parallel 10x system [1]. Each model was able to find three distinct regions based on expression patterns (Fig. 6). PCA is dominated by total counts, with cells with more reads moving further away in latent space, and more frequent cell types dominating the space [36]. While all methods place high-expression cells together, nonlinear methods like tGPLVM and scVI disperse low-count cells across the space more than linear methods (Fig. 6). We see visually that tGPLVM identifies three distinct regions while providing continuity between cells, so that gene expression is not partitioned into distinct groups in the latent space. CD34 is a marker for hematopoeitic stem cells [37], which differentiate into myeloid and lymphoid cells. From tGPLVM, we can observe this separation from different expression patterns in progenitor cells across dimensions. Dimension three correlates with myeloid cells, demonstrated visually by marker *TYROBP* [38] (Pearson’s *r*=0.647; Fig. 6), in addition to correlations with macrophage-associated genes [39, 40] *S100A4* (Pearson’s *r*=0.623) and *S100A6* (Pearson’s *r*=0.665). In comparison to the traditional GPLVM, tGPLVM collects cells expressing *TYROBP* more compactly (Fig. 6). Dimension two correlates to lymphoid cells, visualized by marker *LTB* [41] (Pearson’s *r*=0.306; Fig. 6), and further supported by correlation with lymphocyte specific protein-1 *LSP1* (Pearsons’s *r*=0.481). Dimension one corresponds to general cellular functions, with strong correlation with mitochondrial activity genes *COX5A* (Pearson’s *r*=0.587) and *STOML2* (Pearson’s *r*=0.474), and shown with *CLTA*, an endocytosis-mediating gene [42] (Pearson’s *r*=0.461; Fig. 6). These distinct expression patterns reflect the broadly different immune cellular functions into which hematopoietic stem cells may develop. Gradients of expression levels projected onto tGPLVM embeddings may be used to further interrogate changes in cell states from different experiments.

### tGPLVM scales to a million cells

**Fig. 7:**
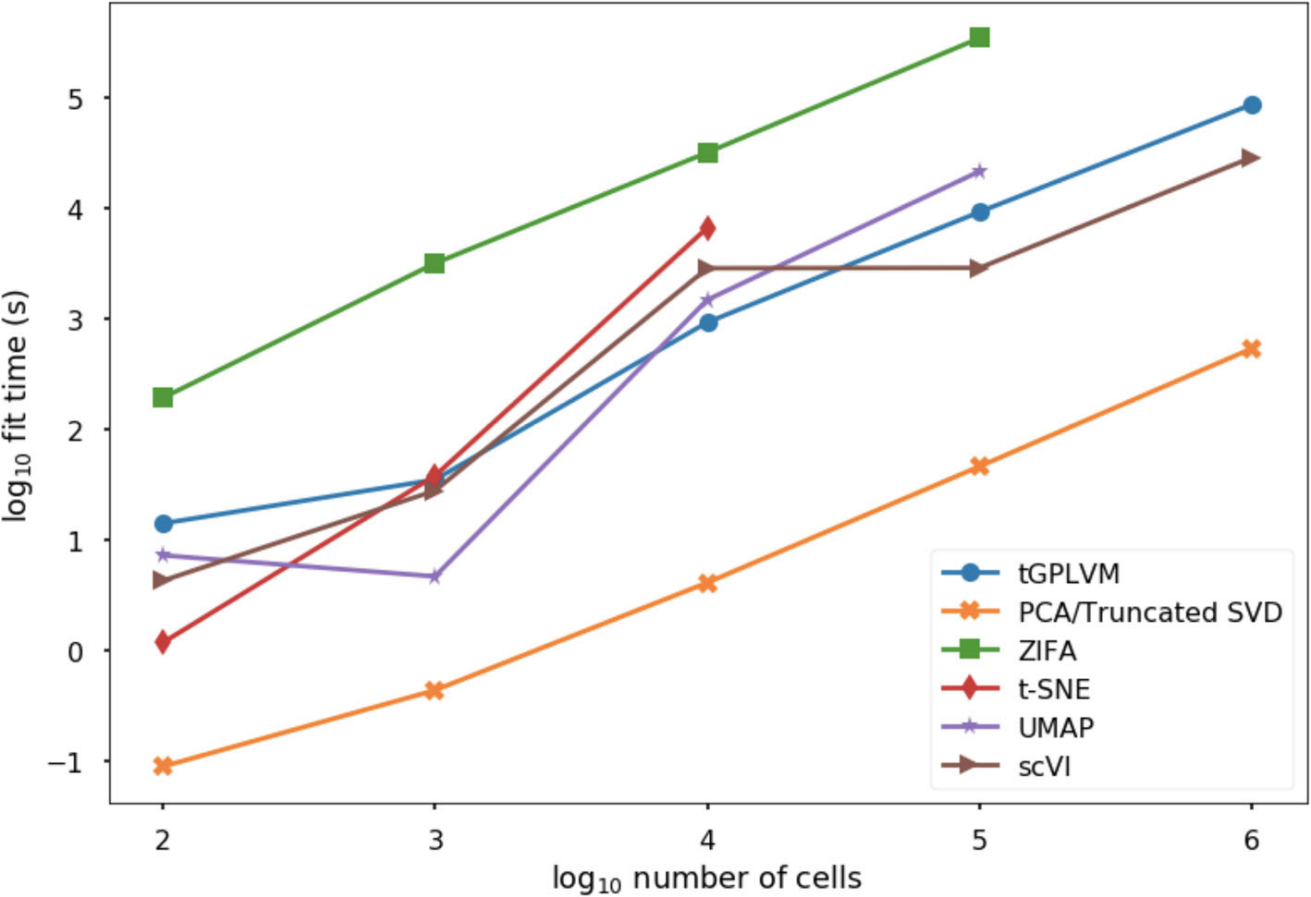
Time to fit a two dimensional embedding vs. sample size on a 16 VCPU, 224 GB memory high performance computing system

Finally, we evaluate the ability of tGPLVM and related methods to fit embeddings for unfiltered, unnormalized, high throughput scRNA-seq data. Models with two latent dimensions were fit on subsamples from 100 to 1 million cells from the 10x 1 million mouse brain cell data [1]. tGPLVM, PCA, and scVI are the only methods that can fit one million cells in a computationally tractable way (Fig. 7). ZIFA is slower than tGPLVM by an order of magnitude consistently across sample sizes. Since ZIFA requires a dense input, its input cell count matrix is limited in our framework to approximately 100,000 cells. While t-SNE’s implementation can take as input a sparse matrix format, it does not converge beyond 10^4^ samples. Similarly, UMAP is not able to fit more than 10^5^ cells.

**Fig. 8:**
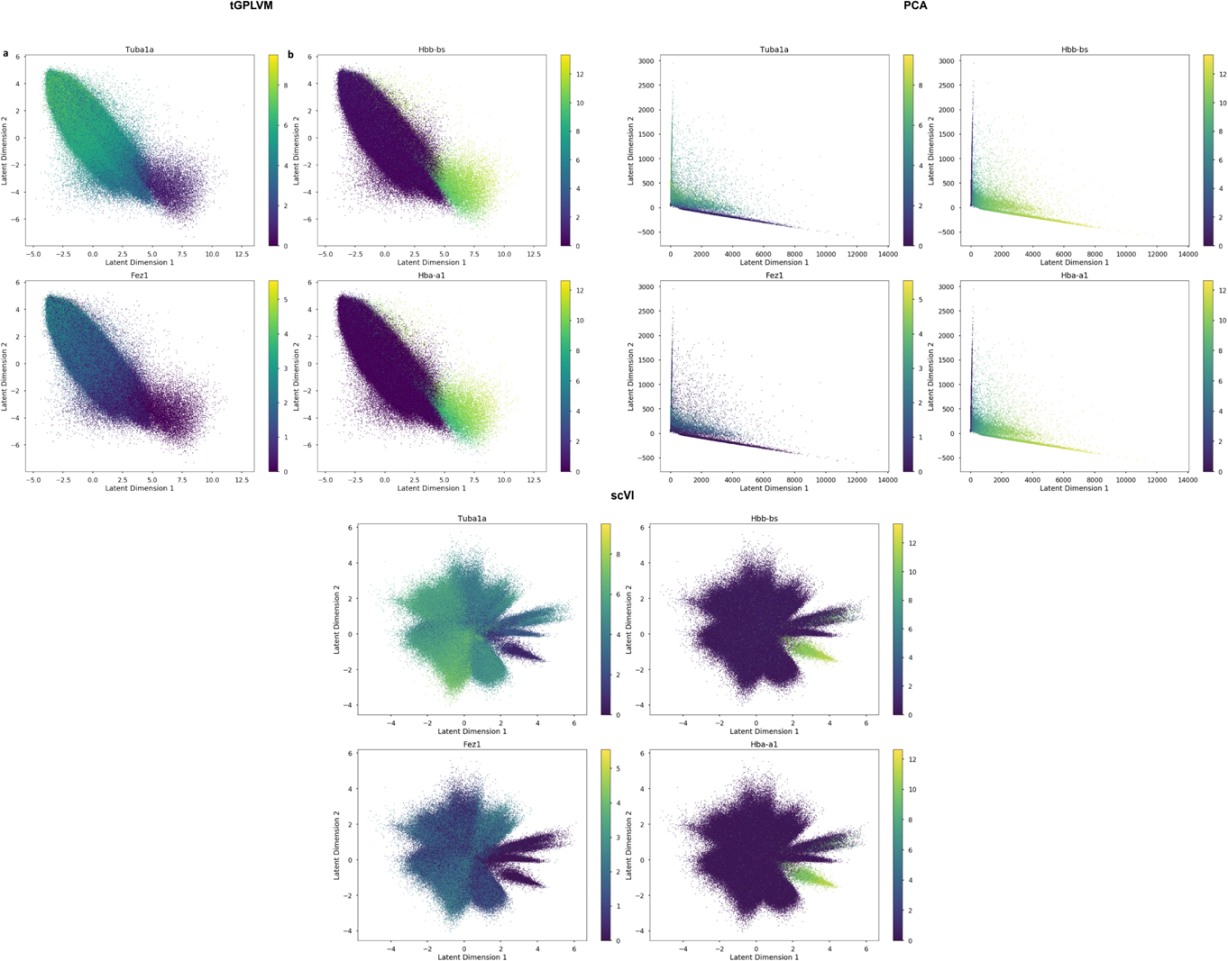
Gene expression patterns across tGPLVM, PCA, and scVI manifolds in 1 million mouse brain cells [1]. Latent dimension one separates circulatory and blood genes. Latent dimension two is correlated with neural genes. Color bars indicate log2(1+*Y*), where *Y* represents total counts per gene per cell

To check that the embedding has biological significance, we again used Pearson’s correlation to identify genes whose expression is correlated with latent dimensions. We find that latent dimension one corresponds to increased expression of genes associated with the circulatory system and hemoglobin, such as *HBB-BS* (Pearson’s *r*=0.320) and *HBA-A1* (Pearson’s *r*=0.316; Fig. 8b). Dimension two correlates with genes such as *TUBA1A* (Pearson’s *r*=0.474) and *FEZ1* (Pearson’s *r*=0.427) that are associated with neural cells (Fig. 8a). While scVI and PCA also find similar separation, visually tGPLVM provides a clear gradient between the patterns (Fig. 8). The ability of tGPLVM to scale to high-throughput data and capture global structure from unnormalized count matrices makes it a powerful method for analyzing upcoming large-scale single-cell experiments.

## Discussion

Our results show that tGPLVM is flexible to cell type, cell development, and cell perturbation experiments and finds information-rich mappings from filtered and processed data as well as unfiltered raw count data. tGPLVM scales to the size of a million cells as produced by the latest single-cell sequencing systems. Despite the sparsity, these data are complex and require more than two factors to capture variation; we did not use the ARD kernel parameters to remove dimensions for any of our experiments. However, the embedded dimensions in our experiments were able to capture informative representations of these complex data. As we increased the number of latent dimensions, at some point the ARD prior would remove unnecessary dimensions for being redundant.

We expect that this general statistical model of dimension reduction may be built upon for more sophisticated, nonparametric approaches for a variety of single-cell tasks from normalization and imputation to cell type identification and pseudotime inference. The optimal method for modeling space count data is an open area of research. Alternative models could explore the use of Poisson, negative binomial, or multinomial emission models. While the t-distribution is robust to different distributions, there may be other transformations of the count data that better stabilize variance or reduce heteroskedasticity.

## Conclusion

We present a Bayesian nonparametric model for robust nonlinear manifold estimation in scRNA-seq settings. tGPLVM captures transcriptional signals in single-cell data using a robust Student’s t-distribution noise model and integrating adaptive kernel structure in settings with no *a priori* information about clusters or sequencing order. We hope that this robust manifold estimation can be used for other types of data with noisy outliers and sparse features.

## Methods

### The t-distribution Gaussian process latent variable model (tGPLVM).

The tGPLVM assumes that samples in high-dimensional space are noisy observations of a Gaussian process of lower-dimensional latent features. Let $Y \in \mathbb {R}^{N\times P}$ represent *N* observations in a high-dimensional space of dimension *P*, and let $X \in \mathbb {R}^{N \times Q}$ represent the same observations in a lower-dimensional space *Q*≪*P*. Each sample *x*_*n*_ in *n*∈{1,2,...,*N*} is assumed to be drawn from a *Q* dimensional multivariate normal distribution with identity variance:

$$ x_{i} \sim \mathcal{N}_{Q} (0, I_{Q}). $$

Noiseless observations of each of the *P* high-dimensional features across *N* samples, *f*_*p*_(*X*), are draws from a zero-mean Gaussian process of *x* across a weighted sum of *M* kernels:

$$\begin{array}{@{}rcl@{}} f_{p}(X) & \sim& \mathcal{N}_{n} (0, K_{NN}) \\ k(x,x^{\prime}) &=& \sum_{m=1}^{M} k_{m} (x,x^{\prime}), \end{array} $$

where *K*_*NN*_ represents the *N*×*N* covariance matrix defined by the kernel function, *k*(*x*,*x*^′^). In the traditional GPLVM, observations *y*_*n*,*p*_ are noisy realization of a normal distribution with mean *f*_*n*,*p*_ and variance *τ*^2^:

$$\begin{array}{@{}rcl@{}} y_{n,p} | f_{n,p}(X), \tau^{2} &\sim& \mathcal{N}(f_{n,p}(X),\tau^{2}). \end{array} $$

For tGPLVM, each observation *y*_*n*,*p*_ is drawn from a heavy-tailed Student’s t-distribution with a set degrees of freedom *ν* and feature-specific variance $\tau _{p}^{2}$ [28]:

$$\begin{array}{@{}rcl@{}} y_{n,p} | f_{n,p}(X),\tau_{p}^{2},\nu & \sim & \textrm{StudentT}(f_{n,p}, \nu, \tau_{p}^{2})\\ &=& \frac {\Gamma((\nu+1)/2)}{\Gamma(\nu/2)\sqrt{\nu\pi}\tau_{p}} \left(1+ \frac {(y_{n,p} - f_{n,p})^{2}}{\nu \tau_{p}^{2}}\right)^{-(\nu+1)/2}, \end{array} $$

where we use *f*_*n*,*p*_ to represent the *n*th component of the *N* dimensional vector *f*_*p*_(*X*). We set *ν*=4 for expression data based on previous work with Gaussian process regression with t-distributed error [28, 29].

The kernel that we use is a flexible sum of an automatic relevance determination (ARD) squared exponential kernel and three different Matérn ARD kernels, each with hyperparameters scales *σ*_*m*_ and length scales *ℓ*_*m*,*q*_. Each ARD dimension-specific length scale, *l*_*k*,*q*_, indicates the distance of that latent dimension over which points are similar. While nonparametric covariance estimation techniques may be more flexible [43, 44], in this formulation, kernel parameters – and associated kernel shape – are estimated using maximum likelihood methods simultaneously with latent variable estimation. Letting *r* represent the length scale-weighted distance in latent space, the kernels are defined as:

$$\begin{array}{@{}rcl@{}} r_{m} &=& \sum_{q =1}^{Q} \frac{|x_{q} - x^{\prime}_{q}|}{\ell_{m,q}} \\ k_{1}(x,x^{\prime}) &=& k_{SE}(x,x^{\prime}) = \sigma^{2}_{1} \exp\left\{- \frac 1 2 r_{1}^{2} \right\} \\ k_{2}(x,x^{\prime}) &=& k_{Mat 1/2}(x,x^{\prime}) = \sigma^{2}_{2} \exp\left\{-r_{2}\right\} \\ k_{3}(x,x^{\prime}) &=& k_{Mat 3/2}(x,x^{\prime}) = \sigma^{2}_{3} (1 + \sqrt{3}r)\exp\left\{-\sqrt{3}r_{3}\right\} \\ k_{4}(x,x^{\prime}) &=& k_{Mat 5/2}(x,x^{\prime}) = \sigma^{2}_{4} (1 + \sqrt{5}r + \frac{5}{3}r^{2})\exp\left\{-\sqrt{5}r_{4}\right\}. \end{array} $$

We use black box variational inference (BBVI) [45] (see Appendix A) to estimate the posterior distribution for the tGPLVM. We adapt the variational distributions from prior work [46]. Inference is implemented in Python using Edward [47, 48]. Edward optimizes the hyperparameters to maximize the conditional likelihood *p*(*x*|*z*,*λ*). The use of BBVI means there is no computational cost to changing the error model and kernels. However, the switch from exact gradient-based methods of traditional VI for GPLVMs [46] to approximations via BBVI may have an accuracy cost.

To scale to large data, minibatches of cells and genes are used to approximate gradients at each step. Genes (i.e., features) are sampled in proportion to the percentage of cells in which they are expressed to efficiently approximate the covariance matrix calculated during inference, inspired by previous random matrix algorithms for approximate matrix multiplication [49]. Cells (i.e., samples) are sampled uniformly in every batch. Inference was performed on Microsoft Azure High Performance Computing cores.

### Simulation experiments

We simulated ten-dimensional observations from a ground truth two-dimensional branching manifold using Gaussian processes with different kernels and error models. Latent embeddings were estimated for tGPLVM with both composite kernels and normal kernels. We also compared models with a normal error and Student’s t-distributed error with degrees of freedom between 1 and 10. Each test (simulation and inference) was repeated one hundred times. We tested two different noise models with smooth RBF kernels: normally-distributed error with mean zero and variance between 0.1 and 1 times the maximum value of noiseless data, and skewed noise with a gamma-distributed error with scale 1 and shape between 0.1 and 1 times the maximum value of the noiseless data.

To compare the estimated manifolds to ground truth, we used the Wasserstein-2 distance between the row-normalized Euclidean distance matrices between all observations, with a cost matrix equal to the pair-wise distance in the ground truth. Intuitively, this penalizes large distances between points that were nearby in the true latent space.

### Single cell RNA-seq data

We chose four data sets to evaluate tGPLVM’s applicability to identify cell type, state, and developmental trajectory, and scalability to experiments with large numbers of cells. The Pollen data [33], which were used to evaluate clustering, consist of 11 distinct mouse neural and blood cell populations across 249 cells sequenced on a Fluidigm C1 systems. Pollen is a dense matrix because of high read depth, with about 80% non-zero values. The counts, *Y*, are log normalized as *Y*^′^= log10(1+*Y*). Inference of development trajectories was evaluated on the data used to develop the method GPfates from Lonnberg [10]. These data include 408 T helper cells sequenced over 7 days following *Plasmodium* infection on a Fluidigm C1 system. The Lonnberg data are provided as TPM measurements. The data are sparse and normalized by log2(1+*Y*). Cell state was explored on batch of CD34+ peripheral blood mononuclear cells [1] (PBMCs). About 10,000 cells were captured with 10x Cell Ranger sequencing technology. These sparse data were also normalized as log2(1+*Y*). Finally, to ensure scalability to the most recent experimental data sets, we fit the model to 1 million mice brain cells sequenced on a 10x Cell Ranger [1]. We normalized the mice brain cells as log2(1+*Y*).

#### Identifying cell types with k-means clustering

Clustering for cell-type identification was evaluated on the Pollen [33] data. tGPLVM and comparison methods were used to fit latent mappings between 2 and 9 dimensions. To perform clustering for each of the estimated latent manifolds, we used k-means clustering with the number of clusters *k* equal to the number of different cell type or cell state labels in the existing data. Clustering with k-means was repeated ten times on the mean of the maximum *a posterior* estimate of the latent position and evaluated against true labels using normalized mutual information (NMI) and adjusted rand score (ARS). Mutual information measures the amount of information contained about one random variable (the true labels) in another random variable (the inferred labels). NMI normalizes mutual information by the geometric mean of the entropy of both labels to a scale of zero – no mutual information – to one – the same distribution [50]. ARS is a measure of the proportion of shared members between pairs of true and estimated clusters [51]. Zero inflated factor analysis (ZIFA) [4], t-SNE [7] (perplexity set to default 30), UMAP [21] (default settings), scVI [23], and PCA [14] were tested as comparison methods. To evaluate the robust adaptations of the tGPLVM model, we fit tGPLVM with only a SE kernel or SE and Matérn 1/2 kernel, as well as a tGPLVM with normally distributed error.

#### Trajectory building with minimum spanning trees

tGPLVM was used to fit a two dimensional map for the Lonnberg developmental data [10]. The minimum spanning tree was found on the Euclidean distance matrix of the posterior means of the low-dimensional embedding, and compared to sequencing time to verify correct ordering. The same analysis was performed with ZIFA [4], t-SNE [7] (perplexity set to default 30), UMAP [21], scVI [23], and PCA [14].

#### Visualization of sparse, raw count matrices

tGPLVM was used to fit a three-dimensional mapping for the two 10x data sets, CD34+ cells and mice brain cells. Pearson correlation between latent position posterior mean and expression counts was used to identify genes associated with latent dimensions. ZIFA [4], t-SNE [7] (perplexity set to default 30), UMAP [21], scVI [23] and PCA (truncated SVD [52]) were also fit to the CD34+ cell data.

### Scaling inference to high-throughput experiments

Computational times were recorded for samples of 100, 1,000, 10,000, 100,000 and 1,000,000 cells from the 10x 1 million mouse brains cells [1] to a latent embedding using tGPLVM and comparison methods. Experiments were run on a Standard H16m (16 VCPUs, 224 GB memory) Azure high performance computing Unix system. tGPLVM was run for 100 passes through the data. Each mini-batch contained the minimum of 2500 cells or the number of cells, and 250 genes. ZIFA, t-SNE, UMAP, scVI, and PCA (using truncated SVD) were fit until convergence or failure.

## Supplementary information

**Additional file 1** A PDF file with two appendices. Appendix A describes the variational distributions for inference. Appendix B lists the sources of the various data sets and implementations of comparison methods.

**Additional file 2** Supplemental figure 1–sensitivity of clustering results to initialization. The normalized mutual information and adjusted rand score when comparing clusters learned from K-Means applied to tGPLVM fit to true cluster labels when tGPLVM is initialized with PCA or random Gaussian noise.

## Data Availability

The pollen data and implementation is available in the tGPLVM repository (https://github.com/architverma1/tGPLVM). Links to repositories for the remaining data is also available in the tGPVLM repository.

## Abbreviations

GPLVM: Gaussian Process Latent Variable Model
tGPVLM: Student’
s t-distributed Gaussian Process Latent Variable Model; PCA: Principal Component Analysis
t-SNE: t-distributed Stochastic Neighbor Embedding
ZIFA: Zero Inflated Factor Analysis UMAP: Uniform Manifold Approximation and Projection for Dimension Reduction
scVI: Single Cell Variational Inference
NMI: Normalized Mutual Information
ARS: Adjusted Rand Score
