# Supplementary material for: A robust nonlinear low-dimensional manifold for single cell RNA-seq data
Source: BMC Bioinformatics. 2020 Jul 21;21:324. doi: 10.1186/s12859-020-03625-z (PMC7374962; doi:10.1186/s12859-020-03625-z)
Supplement: Supplementary file 1 — Additional file 1 A PDF file with two appendices. Appendix A describes the variational distributions for inference. Appendix B lists the sources of the various data sets and implementations of comparison methods. [file 12859_2020_3625_MOESM1_ESM.pdf]

METHODS

# A robust nonlinear low-dimensional manifold for single cell RNA-seq data

Archit Verma[1*] and Barbara E Engelhardt[2]

*Correspondence: architv@princeton.edu
[1]Chemical and Biological Engineering, Princeton University, Olden Street, 08540 Princeton, NJ, US
Full list of author information is available at the end of the article
†Equal contributor

## Appendix A: Variational Inference

We fit tGPLVM to data with Black Box Variational Inference [1]. BBVI uses sampling to stochastically compute gradients to minimize the KL Divergence, or equivalently maximize the Evidence Lower Bound, between an approximating variational distribution and the true posterior. Gradients of the evidence lower bound (ELBO) do not require gradients of the log probability, avoiding the derivative of the Student's t-distribution.

For GPLVMs, variational inference is implemented by introducing auxiliary variables known as inducing points [2, 3]. Inducing points reduce the complexity of fitting the model from $O(n^3)$ to $O(m^2n)$ [2]. We adapt the variational inference methods from [2] for the Bayesian GPLVM. Maintaining the variables and notation from the model, we introduce inducing points $X_U \in \mathbb{R}^{M\times Q}$, and latent GP evaluations at the inducing points $U \in \mathbb{R}^{N\times P}$. The likelihood for the model can be rewritten as:

$$p(Y, F, U, X|X_u) = \left(\prod_{j=1}^{P} p(y_j|f_j)p(f_j|u_j, X, X_u)p(u_j|X_u)\right)p(X).$$

This is approximated by a variational distribution of form:

$$q(F, U, X) = \left(\prod_{j=1}^{P} p(f_j|u_j, X)q(u_j)\right)q(X).$$

The variational distribution over q(X) is a Gaussian:

$$q(x) = \mathcal{N}(x|\mathcal{M}, \mathcal{S}),$$

where $\mathcal{M}$ and $\mathcal{S}$ are variational parameters for the mean and variance of the posterior. The distribution of $q(U)$ is unconstrained [2]. We use the following formulation:

$$\begin{aligned}
K_{fu} &= k(x, u') \\
K_{ff} &= k(x, x') \\
K_{uu} &= k(u, u') \\
\psi_{uu} &= (K'_{fu}K_{fu})^{-1} \\
q(u) &= \mathcal{N}(u|K_{uu}\psi_{uu}K_{fu}y, K_{uu}).
\end{aligned}$$

The probability of $f_j$ given the latent variables and inducing points is:

$$p(f_j|u_j, X) = \mathcal{N}(f|K_{fu}K_{uu}^{-1}u, K_{ff} - K_{fu}K_{uu}^{-1}K'_{fu}).$$

Variational latent means, $\mathcal{M}$, are initialized with PCA (truncated SVD for sparse format matrices) unless otherwise indicated. Variational latent variances, $\mathcal{S}$, are initialized as ones. The initial inducing points $X_u$ are randomly sampled from the initial latent means. We use 30 inducing points in all experiments.

## Appendix B: Data Acquisition and Implementations

Estimated manifolds were compared to zero inflated factor analysis (ZIFA) [4], t-SNE [5], scVI [6] and PCA [7] (dense data) or TruncatedSVD [8] (sparse data). ZIFA was implemented using available Python code (https://github.com/epierson9/ZIFA). scVI was also implemented using available Python code (https://github.com/YosefLab/scVI). t-SNE was implemented using scikit-learn with the default perplexity of 30. PCA and truncated SVD were also implemented from scikit-learn. For all experiments we also tested our model with different kernels and with a normal, but gene-specific, error model.

The high count expression matrix for Pollen [4] was downloaded from the SIMLR repository (https://github.com/BatzoglouLabSU/SIMLR) [9]. These data are log normalized by $\log_{10}(1+Y)$. The data consist of 249 cells from 11 cell populations. Due to its small size, the full data set was used for each batch. K-means clustering on latent variable mappings was performed using scikit-learn's sklearn.cluster. KMeans [10]. NMI and Rand were computed using scikit-learn's NMI (sklearn.metrics.normalized_mutual_info_score) and Adjusted Rand Score packages (sklearn.metrics.adjusted_rand_score) [10].

The data from GPfates [11] were downloaded from the GPfates repository (https://github.com/Teichlab/GPfates/tree/master/) and includes the TPM normalized expression for 409 *Plasmodium*-infected CD4+ T cells sequenced in batches over the course of seven days. These data were log normalized by $\log_2(1+Y)$. Minibatches included 408 cells and 1700 genes. For each estimated manifold, a minimum spanning tree was fit to the undirected graph matrix of Euclidean distance between the cells' locations in the latent space using. scipy.sparse.csgraph.minimum_spanning_tree.

The filtered count matrix for CD34+ PMBCs [12] was downloaded from the 10x website (https://support.10xgenomics.com/single-cell-gene-expression/datasets/1.1.0/cd34). Minibatches consisted of 1000 cells and 500 genes. Data was log normalized as $\log_2(1+Y)$. Inference was run for 250 iterations. The count matrix of 1 million mouse brain cells [12] was also downloaded from the 10x website (https://support.10xgenomics.com/single-cell-gene-expression/datasets/1.3.0/1M_neurons). Counts were normalized by $\log_2(1+Y)$. Minibatches were sized as described in methods. Due to memory constraints in implementation of TruncatedSVD, the 1 million neural brain cells latent means were initialized using nonnegative matrix factorization (NMF) [13]

**Author details**
[1]Chemical and Biological Engineering, Princeton University, Olden Street, 08540 Princeton, NJ, US. [2]Computer Science, Center for Statistics and Machine Learning, Olden Street, 08540 Princeton, NJ, USA.

**References**

1. Ranganath, R., Gerrish, S., Blei, D.M.: Black Box Variational Inference. International Conference on Artificial Intelligence and Statistics (AISTATS) **33** (2013). 1401.0118
2. Damianou, A.C., Titsias, M.K., Lawrence, N.D.: Variational inference for latent variables and uncertain inputs in Gaussian processes. Journal of Machine Learning Research **17**, 1–62 (2016)

3. Titsias, M., Lawrence, N.: Bayesian Gaussian process latent variable model. Artificial Intelligence **9**, 844–851 (2010). 1309.6835
4. Pierson, E., Yau, C.: ZIFA: Dimensionality reduction for zero-inflated single-cell gene expression analysis. Genome Biology **16**(1), 241 (2015)
5. Van Der Maaten, L.J.P., Hinton, G.E.: Visualizing high-dimensional data using t-SNE. Journal of Machine Learning Research **9**, 2579–2605 (2008). 1307.1662
6. Lopez, R., Regier, J., Cole, M.B., Jordan, M.I., Yosef, N.: Deep generative modeling for single-cell transcriptomics. Nature methods **15**(12), 1053–1058 (2018)
7. Hotelling, H.: Analysis of a complex of statistical variables into principal components. Journal of Educational Psychology **24**(6), 417 (1933)
8. Halko, N., Martinsson, P.-G., Tropp, J.A.: Finding structure with randomness: Probabilistic algorithms for constructing approximate matrix decompositions. arXiv preprint arXiv:0909.4061, 1–74 (2009)
9. Wang, B., Zhu, J., Pierson, E., Ramazzotti, D., Batzoglou, S.: Visualization and analysis of single-cell RNA-seq data by kernel-based similarity learning. Nature Methods **14**, 414 (2017)
10. Pedregosa, F., *et al.*: Scikit-learn: Machine learning in Python. Journal of Machine Learning Research **12**, 2825–2830 (2011)
11. Lönnberg, T., *et al.*: Single-cell RNA-seq and computational analysis using temporal mixture modeling resolves TH1/TFH fate bifurcation in malaria. Science Immunology **2**(9), 2192 (2017)
12. Zheng, G.X.Y., *et al.*: Massively parallel digital transcriptional profiling of single cells. Nature Communications **8**, 14049 (2017)
13. Lee, D., Seung, H.: Algorithms for non-negative matrix factorization. Advances in Neural Information Processing Systems (1), 556–562 (2001). 0408058v1
