# Supplementary material for: A robust nonlinear low-dimensional manifold for single cell RNA-seq data
Source: BMC Bioinformatics. 2020 Jul 21;21:324. doi: 10.1186/s12859-020-03625-z (PMC7374962; doi:10.1186/s12859-020-03625-z)
Supplement: Supplementary file 2 — Additional file 2 Supplemental figure 1–sensitivity of clustering results to initialization. The normalized mutual information and adjusted rand score when comparing clusters learned from K-Means applied to tGPLVM fit to true cluster labels when tGPLVM is initialized with PCA or random Gaussian noise. [file 12859_2020_3625_MOESM2_ESM.pdf]

1 Supplementary Figures

1.1 Effect of Initialization on Clustering

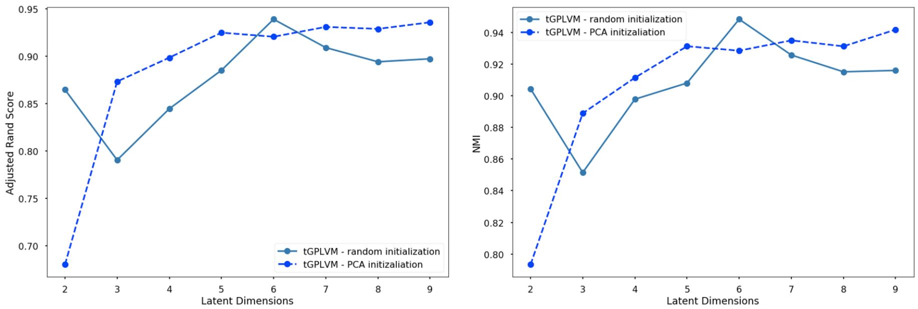

Figure 1: Black Box Variational Inference can be sensitive to initialization. As evaluated by NMI and Adjusted Rand Score we find that changing from a PCA initialization to random noise initialization does not greatly change clustering performance.
